# An Uncommon Mould in an Uncommon Place: A Case of Scopulariopsis brevicaulis Keratitis From the Northern Himalayas, India

**DOI:** 10.7759/cureus.106303

**Published:** 2026-04-01

**Authors:** Pratiksha Kamboj, Amber Prasad, Sowjanya Perumalla, Binal Mangroliya, Tanisha Sharma

**Affiliations:** 1 Microbiology, Government Medical College, Haridwar, Haridwar, IND; 2 Microbiology, All India Institute of Medical Sciences, Rishikesh, Rishikesh, IND

**Keywords:** fungal keratitis, natamycin, scopulariopsis brevicaulis, soil fungi, voriconazole

## Abstract

*Scopulariopsis brevicaulis* is an uncommon cause of fungal keratitis, usually occurring after ocular trauma, and is associated with therapeutic difficulties because of its limited susceptibility to antifungal agents. We report the case of a 48-year-old man who presented with pain, redness, and watering of the left eye for one month following injury by iron foreign body particles during welding. Ocular examination revealed a corneal ulcer with hypopyon and markedly reduced visual acuity (2/60). Initial microscopic evaluation was negative for fungal elements; however, fungal culture after extended incubation yielded growth of *S. brevicaulis*, identified based on colony characteristics and microscopic morphology. Imaging further demonstrated left-sided dacryoadenitis. The patient was treated conservatively with intensive topical natamycin and voriconazole, along with systemic ketoconazole and supportive topical antibiotics. This approach led to clinical improvement, although some degree of visual impairment persisted. This case highlights the importance of maintaining a high index of suspicion, allowing prolonged incubation of fungal cultures, and initiating timely targeted antifungal therapy in rare mould keratitis to achieve favorable outcomes and potentially avoid surgical intervention.

## Introduction

Mycotic keratitis is a major contributor to vision-related morbidity across the globe, with the burden being especially high in tropical and subtropical regions, where it accounts for 20-60% of all culture-positive corneal infections [1]. Early diagnosis remains challenging due to overlapping clinical features with bacterial keratitis, and delayed or inappropriate treatment frequently results in irreversible corneal scarring and blindness [1,2].

Over 50 distinct fungal species have been implicated as causative agents, with *Fusarium* species being the most prevalent, followed by *Aspergillus* species [3]. However, rare and less frequently encountered moulds are increasingly being recognised as pathogens, particularly following ocular trauma or in atypical clinical settings [2].

*Scopulariopsis* species are uncommon opportunistic pathogens known to cause localised infections, including keratitis, onychomycosis, and otomycosis [4]. Among them, *Scopulariopsis brevicaulis* is an exceptionally rare cause of fungal keratitis, with only a handful of cases described in the literature worldwide. This report presents one such rare case from the Northern Himalayas, highlighting the importance of considering uncommon fungal pathogens in the differential diagnosis of corneal infections.

## Case presentation

**Figure 1:**
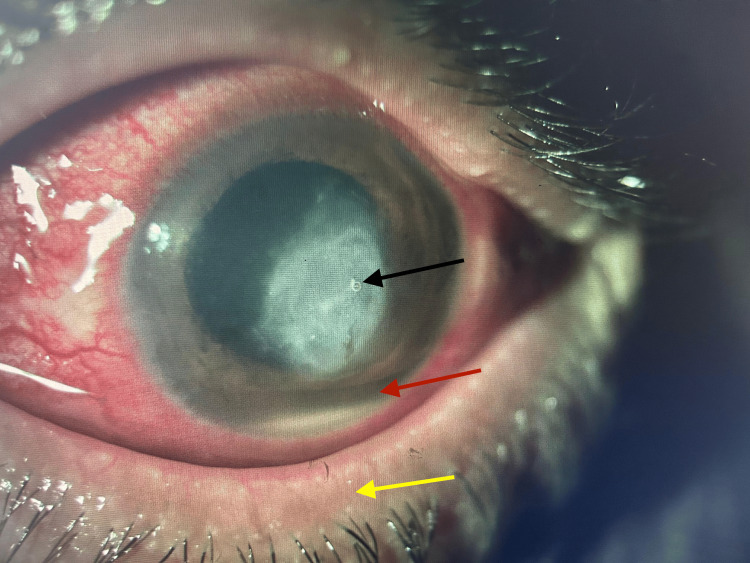
Slit-lamp photo of left eye showing a fungal corneal ulcer. The image demonstrates a central corneal ulcer (black arrow) with surrounding stromal infiltrate, associated hypopyon (red arrow), and lid oedema with congestion (yellow arrow).

A 48-year-old male presented to the ophthalmology outpatient clinic on 25 February 2025 with a one-month history of pain, redness and watery left eye. The patient has a history of trauma due to iron foreign body particles (FB) from a welding machine one month back. On examination, visual acuity was 6/6 (right eye) and 2/60 (left eye). The left eye showed a corneal ulcer with hypopyon, along with lid oedema and congestion (Figure 1).

There was no history of diabetes and hypertension.

A provisional diagnosis of infectious keratitis was made. On the day of presentation (25 February 2025), corneal scraping was performed under aseptic precautions and inoculated onto blood agar, chocolate agar, and Sabouraud dextrose agar. Smears were prepared for Gram stain and a 10% potassium hydroxide (KOH) mount. Gram stain showed numerous pus cells with no organisms, and KOH mount did not reveal fungal elements.

The patient took over-the-counter medication with some unspecified eye drops; however, no documentation was available. The current regimen included fortified cefazolin and tobramycin eye drops hourly. The patient was also receiving oral acetazolamide (Diamox) twice daily. The patient reported symptomatic improvement. The importance of strict compliance with the prescribed medication regimen was clearly explained. It was communicated that some degree of scarring and diminution of vision (DOV) may persist. The overall prognosis was discussed in detail. For pain management, Zerodol-SP tablets were prescribed on an as-needed (SOS) basis.

A computed tomography (CT) scan performed on 3 March 2025 at an outside facility showed that the left lacrimal gland measured 26 × 10 mm. It was enlarged in size as compared to the right, which measured 17 x 6.0 mm. There was no calcification or solid areas seen within it. A non-contrast CT orbit study showed features of dacryoadenitis on the left side. The CT scan of the orbit was performed at an outside facility prior to presentation. At the time of evaluation in our centre, only the official radiology report was available, and the original imaging films/images could not be retrieved.

**Figure 2:**
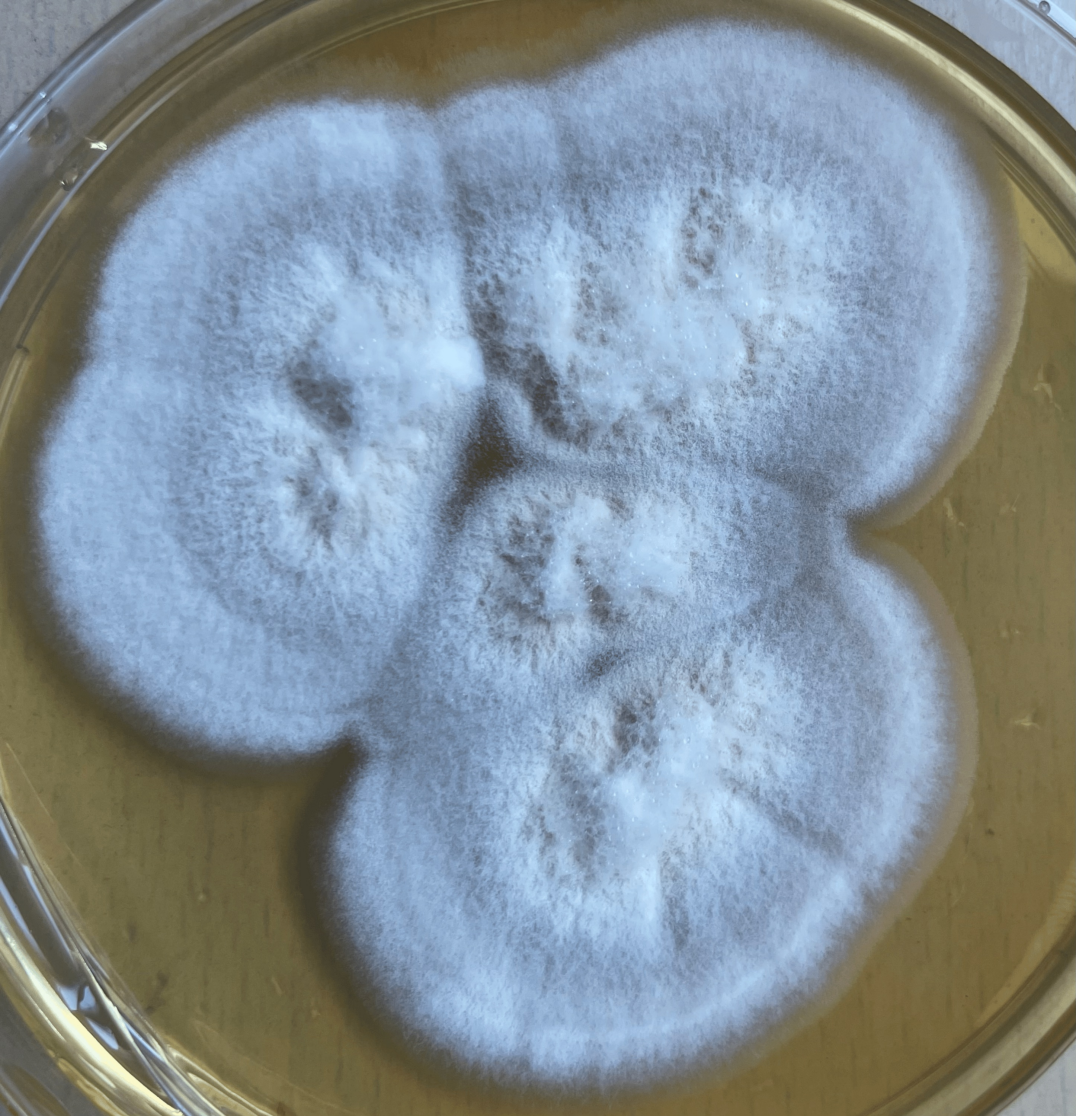
Culture of Scopulariopsis brevicaulis on Sabouraud dextrose agar. Cream-colored colonies with a powdery surface and feathery margins were observed after incubation at 25°C for 10 days, consistent with the characteristic colony morphology of *Scopulariopsis* species.

The inoculated bacterial agar plates of blood and chocolate culture media showed no bacterial growth after 48 hours of aerobic incubation at 37°C (Figure 2).

**Figure 3:**
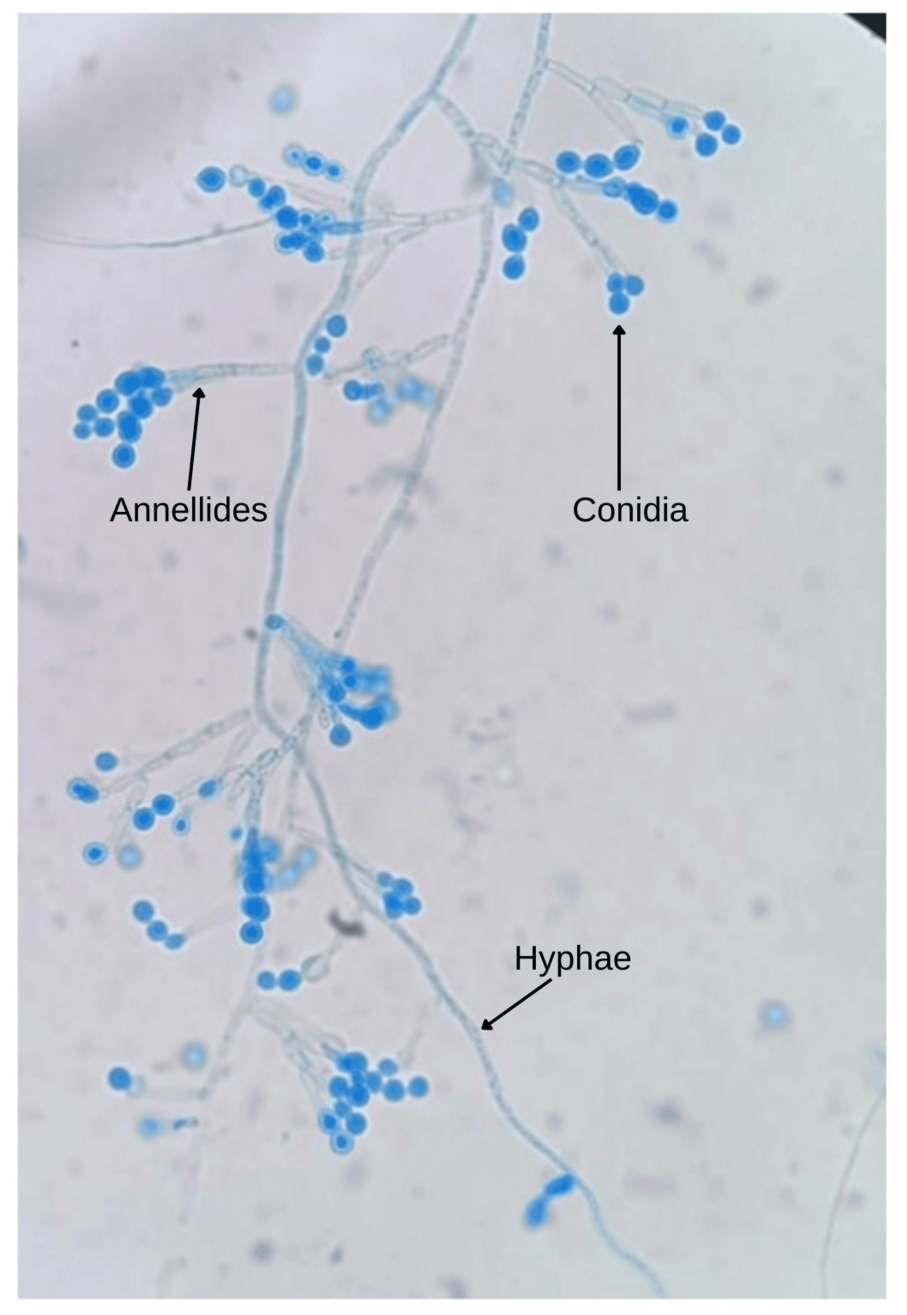
Lactophenol cotton blue mount showing the microscopic morphology of Scopulariopsis brevicaulis (x40). Hyaline septate hyphae, along with numerous annellides and globose conidia, were seen, consistent with the morphological features of *S. brevicaulis*.

However, fungal growth was detected on blood, chocolate and Sabouraud dextrose agar after 10 days of aerobic incubation at 37°C, in which cream-coloured colonies with powdery surface and feathery borders were seen. On microscopic examination of colonies stained with lactophenol cotton blue, hyaline septate hyphae, numerous annellides and globose-shaped conidia were observed (Figure 3).

The patient was prescribed natamycin 5% eye drops (Natamet) to be instilled hourly in the left eye until bedtime. Oral ketoconazole 200 mg (Ketocip) was prescribed twice daily, and pantoprazole with domperidone (Pantop-D) once daily. Atropine sulfate 1% eye drops were administered three times daily in the left eye. Voriconazole 1% eye drops (Vozole) were prescribed six times daily in the left eye. Moxifloxacin 0.5% eye drops (Vigamox) were initially administered every two hours in the left eye until bedtime for a total duration of 14 days. Following confirmation of the provisional diagnosis, moxifloxacin was discontinued. Identification of *S. brevicaulis* was based on characteristic colony morphology and microscopic features on lactophenol cotton blue mount. Treatment was continued with topical 1% voriconazole alone for a further 45 days. The patient showed a favourable response to therapy, with a gradual reduction in inflammatory signs and mild residual visual impairment. Following residual corneal scraping, voriconazole was discontinued.

## Discussion

*S. brevicaulis* is a very rare cause of keratitis. As these organisms are soil-dwelling saprophytic fungi [5], the patient’s prior ocular trauma with iron FB particles a month earlier likely served as the portal of infection. Only a limited number of keratitis cases caused by *S. brevicaulis* have been documented globally, typically occurring in association with ocular trauma or pre-existing corneal pathology.

Similar to our study, Lotery et al. reported a case of *S. brevicaulis* keratitis in a patient who sustained ocular injury from molten lead splashes, which responded well to amphotericin B therapy [6]. Ragge et al. reported a 26-year-old male who developed progressive keratitis with hypopyon after ocular injury from a nail embedded in decaying wood. The infection was unresponsive to systemic and topical amphotericin B with adjunct itraconazole, ultimately requiring emergency keratoplasty [7]. Malecha described a severe case of *S. brevicaulis* keratitis in a 28-year-old farmer following corneal perforation from a wire injury to the right eye, which responded favourably to natamycin therapy [8]. A case of *S. brevicaulis* infection was previously reported from India by Ghosh et al. [9]. Retrospective review spanning six years from northern India further reported two cases involving *Scopulariopsis* species, underscoring the rarity but recurring presence of this pathogen [10].

The marked resistance of these fungi to most antifungal agents, including amphotericin B and voriconazole, commonly employed for prophylaxis and initial management of systemic mould infections, is a major concern. An effective standard therapy for *Scopulariopsis* infections remains undefined [11]. Management of fungal keratitis typically demands extended antifungal therapy, and clinicians should remain vigilant for potential relapse despite complete clinical recovery [12]. While complicated cases may necessitate keratoplasty, early or less severe cases often respond well to conservative management and may not require surgical intervention.

The presence of dacryoadenitis in this case was noted on imaging; however, in the absence of microbiological correlation, a direct association with fungal infection could not be established.

A limitation of this report is that the identification of *S. brevicaulis* was based on phenotypic methods, including colony morphology and lactophenol cotton blue microscopy, without molecular confirmation. While these methods are widely used in routine diagnostic settings, molecular techniques could provide more definitive species-level identification.

## Conclusions

While fungal keratitis due to *S. brevicaulis* is uncommon, with limited reports from India and the northern Himalayan region, it represents a serious complication associated with a significant risk of visual impairment. This report emphasises that early clinical suspicion, rapid microbiological diagnosis, and appropriate conservative therapy can achieve satisfactory outcomes. However, as this observation is based on a single case, the therapeutic response may not be universally applicable, particularly in view of the known variability in antifungal susceptibility of *Scopulariopsis* species.
